# The role of the rhinostomy ostium size on functional success in dacryocystorhinostomy

**DOI:** 10.1016/j.bjorl.2021.03.006

**Published:** 2021-04-10

**Authors:** Mümtaz Taner Torun, Ebru Yılmaz

**Affiliations:** a Bandırma State Hospital, Otolaryngology Department, Balıkesir, Turkey; b Bandırma State Hospital, Ophthalmology Department, Balıkesir, Turkey

**Keywords:** Dacryocystorhinostomy, Rhinostomy ostium, Functional success, Punctum lavage

## Abstract

**Introduction:**

Endonasal and external dacryocystorhinostomy procedures have both been used for the treatment of post-saccular obstruction of the lacrimal system. Functional success of these surgeries depends on several factors.

**Objective:**

To evaluate the status of the rhinostomy ostium with endonasal and external approaches in dacryocystorhinostomy operations and to determine the effect of ostium size on postoperative functional success.

**Methods:**

The charts of the patients operated in our hospital between May 2017 and January 2019 were analyzed retrospectively (ethical approval number: 2018–12.04). The patients that were operated in the ophthalmology and otolaryngology departments were included in the study. Endoscopic rhinostomy ostium measurements, punctum lavage findings and complications were recorded at 8 weeks postoperative at the earliest.

**Results:**

When the 64 patient charts were reviewed (76 operations), the mean ostium width was 1.85 ± 1.11 mm in the endonasal approach group and 3.60 ± 2.24 mm in the external approach group. The mean ostium areas in endonasal and external group were 14.61 ± 16.66 mm^2^ and 56.05 ± 60.41 mm^2^, respectively. The ostium was anatomically patent and punctum lavages were negative in 11 patients (6 patients in the endonasal approach group and 5 patients in the external approach group) and these cases were considered as functional failures. The rhinostomy ostium was significantly wider in the external approach group, but this was considered ineffective on functional outcomes.

**Conclusion:**

Lacrimal duct stenosis can be successfully treated with endonasal and external methods. Tear drainage may be insufficient even in the presence of a patent ostium. Therefore, functional success should also be considered when evaluating the overall success of dacryocystorhinostomy. An anatomically patent ostium is definitely required, while it is believed that ostium size does not affect functional surgical success.

## Introduction

Epiphora is a health problem that affects the daily life of the patient and causes social embarrassment. Functional occlusions, punctum stenosis, canalicular obstructions and nasolacrimal obstructions are among the most common causes of epiphora.^1^ These can be congenital as well as caused by acquired conditions including trauma, inflammation, and tumors. The aim of nasolacrimal duct obstruction treatment is to restore the drainage of tears into the nose. Dacryocystorhinostomy (DCR) is used in the treatment of post-saccular obstructions. The external approach involves a skin incision, removal of the lacrimal bone and anastomosis of the lacrimal sac to the nasal cavity. The endonasal approach involves the elevation of the lateral nasal wall mucosa and establishment of a fistula by creating a hole using devices such as a laser, power drill, or rongeur.^2^, ^3^, ^4^ Endonasal and external techniques aim to allow the drainage of tears into the nasal cavity by creating a rhinostomy.

There is no consensus on the ideal ostium size in DCR. It is known that rhinostomy ostium becomes smaller in the postoperative period. Studies report that ostium healing is completed by the end of the 4th postoperative week and no significant decrease occurs in the ostium size thereafter.^1^, ^5^, ^6^ Several reasons affect the functional results of the operation, such as postoperative granulation, synechia, wound healing disturbances and infections leading to narrowing of rhinostomy. It is reported that performing a nasal mucosal flap does not affect the success rates of endonasal DCR.^7^ Conflicting results are reported on the effect of the width, shape, size and localization of the ostium on the functional outcomes of DCR operation.

The evaluation of the success of DCR operations has not yet been adequately standardized. Functional results may be poor after an operation that appears to be anatomically successful (e.g., a patent and wide rhinostomy). Our study aimed to assess the effect of ostium size on functional surgical success with both endonasal and external DCR.

## Methods

A retrospective study was planned and ethics approval was granted by the local ethics committee (Approval number 2018-12.04). Sixty-four charts of the operated patients (76 operations) in the Ear–Nose–Throat (ENT) and Ophthalmology Departments between May 2017 and January 2019 were included in the study. The patient charts were reviewed from their hospital files. Age, gender, systemic diseases, surgical notes, postoperative endoscopic nasal examination findings and complication status of the patients were recorded. In our hospital, the patients with the symptoms of lacrimal duct stenosis were examined by an ENT surgeon and an ophthalmologist before the surgery. A 2.7-mm diameter nasal endoscope (Hopkins II telescope, Karl Storz, Tuttlingen, Germany) is used for preoperative and postoperative nasal endoscopic examinations in the ENT clinic. The patients with negative punctum lavage and negative Jones test and the patients older than 18-years old were included in the study. The patients who were operated by the same surgeons (endonasal approach patients by the same ENT surgeon and external approach patients by the same ophthalmologist) were also included in the study to avoid the effect of the surgical technique on the results. The patients who did not complete the postoperative control visits at eight weeks and the revision cases were excluded. Also, the patients who were under 18 years old and had a systemic disease that affected wound healing, such as diabetes mellitus, were excluded. No lacrimal duct probe was used at the end of the both surgeries.

Patients were divided into 2 groups. Group 1 consisted of 38 endonasal operations (6 of them were bilateral operations) and group 2 consisted of 38 external operations (6 of them were bilateral operations). Preoperative punctum lavages and Jones tests of the patients were negative and no preoperative radiological evaluation was performed. The absence of transition to the nasal cavity and the reflux to the other punctum after the application of saline was evaluated as a negative lavage. If the saline appeared in the nasal cavity, it was evaluated as a positive lavage. The earliest ostium measurement was performed after the 8th week by a rigid endoscopic examination, postoperatively. A 4 mm olive-tipped suction was used to measure the vertical and the horizontal diameter of ostium via nasal endoscopy. The functional situation of the duct was controlled by a Jones test at postoperative 4th and 8th week in our clinic.

Granulation and/or synechia around the ostium were treated by the endonasal approach if they were caused an obstruction, postoperatively. In these patients, ostium measurements were performed at 8 weeks following the granulation and synechia treatment. Anatomical success was assessed based on ostium patency. Functional failure was defined as a negative punctum lavage and persistence of epiphora in the presence of a patent ostium.

### Operative techniques

Under general anesthesia, the transnasal endoscopic procedure was performed for endonasal surgeries. After the mucosal incision, the nasal mucosal flap was preserved. The bony window was made with a power drill. A probe was inserted from any punctum to identify the medial wall of the lacrimal sac. The lacrimal sac was opened and flaps were placed to the lateral nasal wall.

Under general anesthesia, a medial canthal incision was performed and the periosteum of the anterior lacrimal crest was dissected in external operations. The lacrimal sac was dissected from the lacrimal fossa and a bony window was opened with a rongeur. After incising the lacrimal sac and nasal mucosa, anterior and posterior flaps of the lacrimal sac were sutured to the nasal mucosal flap.

### Statistical analysis

The data were analyzed by using IBM SPSS Statistics 23.0 software (Armonk, NY: IBM Corp). Frequency, mean and standard deviation were used to analyze the data. The Kolmogorov–Smirnov goodness-of-fit test was used for the normality analysis of the data. *t*-test, Chi-Square and Fisher’s exact test were used to compare the means of the 2-groups since the data showed normal distribution. In multivariate analyzes, multivariate binary logistic regression analyzes with the enter method was used to examine the relationship with functional failure of the ostium width and area in the endonasal and external groups. Functional success status (Functional success: 0, Functional failure: 1) dependent variables, ostium width (continious) and ostium area (continuous) independent variables (Models 1, 2 and 3), age (continious) and gender (Female: 0, Male: 1) were modeled as covariates (Model 3). A *p*-value of <0.05 was considered statistically significant for all analyzes.

## Results

A total of 76 operations was included in the study, of which 38 were endonasal and 38 were external procedure. In the endonasal group, 12 patients were male (31.6%) and 24 patients were female (68.4%) and the mean age was 58.71 ± 11.42. In the external group, 7 patients were male (18.4%) and 31 patients were female (81.6%) and the mean age was 58.42 ± 14.75. The patients were statistically similar in terms of age and gender (Table 1). The shapes of the ostium were circular or oval. The mean ostium width was 1.85 ± 1.11 mm in the endonasal group and 3.60 ± 2.24 mm in the external group. The mean ostium area in endonasal and external group was 14.61 ± 16.66 mm^2^ and 56.05 ± 60.41 mm^2^, respectively. The ostium was statistically significantly wider in the external group (*p* = 0.001). According to multivariate logistic regression analyzes in the endonasal group, it was determined that there was no statistically significant relationship between the functional success of the ostium width and area in Model 1, Model 2 and Model 3 (Table 2). Similarly, in the external group, it was determined that there was no statistically significant relationship between the functional success of the ostium width and area in Model 1, Model 2 and Model 3 (Table 3).

**Table 1:** Patient demographics.

	Endonasal	External	Significance level
Age (mean ± SD)	58.71 ± 11.42	58.42 ± 14.75	*t* = −0.756
			*p* = 0.452^a^
Gender			
Female, n (%)	26 (68.4 %)	31 (81.6 %)	*p* = 0.145^b^
Male, n (%)	12 (31.6 %)	7 (18.4 %)	

a *t*-test.

b Chi-square test.

**Table 2:** Relationship with functional success status of ostium width and area in the endonasal group according to multivariate logistic regression analysis.

	B	SE	OR (95% CI)	*p*
Model 1				
Ostium width	0.393	1.474	1.483 (0.083–26.638)	0.789
Ostium area	−0.018	0.096	0.982 (0.814–1.187)	0.854
Model 2				
Ostium width	0.442	1.483	1.556 (0.085–28.453)	0.766
Ostium area	−0.027	0.099	0.973 (0.801–1.182)	0.783
Model 3				
Ostium width	0.581	1.496	1.789 (0.096–33.577)	0.698
Ostium area	−0.039	0.102	0.962 (0.788–1.174)	0.702

Patients with ostium stenosis (n = 1) were not included in the analysis.

Covariates: age (continious), sex (female:0, male:1).

Model 1, unadjusted; Model 2, adjusted for age; Model 3, adjusted for age and sex.

**Table 3:** Relationship between ostium width and area with functional success in external group according to multivariate logistic regression analysis.

	B	SE	OR (95% CI)	*p*
Model 1				
Ostium width	−1.593	2.691	0.203 (0.001–39.665)	0.554
Ostium area	0.022	0.177	1.022 (0.723–1.447)	0.900
Model 2				
Ostium width	−1.946	2.669	0.143 (0.001–26.702)	0.466
Ostium area	0.040	0.154	1.041 (0.769–1.407)	0.797
Model 3				
Ostium width	−4.346	3.992	0.013 (0.001–32.406)	0.276
Ostium area	0.136	0.158	1.146 (0.840–1.564)	0.389

Patients with ostium stenosis (n = 3) were not included in the analysis.

Covariates: age (continious), sex (male:0, female:1).

Model 1, unadjusted; Model 2, adjusted for age; Model 3, adjusted for age and sex.

In the endonasal group, 20 patients had no complications, while 8 patients had synechia in the nasal cavity (Two were at the level of the ostium and had been removed postoperatively), 8 patients had granulation tissue around the ostium (One of them was excised surgically and 7 of them were treated with medical treatment), 1 patient had a corneal abscess (was healed with medical treatment) and 1 patient had ostium stenosis. In the external group, 27 patients had no complications, while 3 patients had synechia in the nasal cavity (Two of them were at the ostium level and had been removed postoperatively), 5 patients had granulation tissue around the ostium (one of them was excised with endonasal surgical intervention and 4 of them regressed with medical treatment) and 3 patients had ostium stenosis. When both groups were compared in terms of complications, no statistically significant difference was found (*p *= 0.078). Ostium stenosis and punctum lavages were negative in 1 patient in the endonasal group and in 3 patients in the external group and these cases were considered as anatomical failures. Ostium width, complication status and punctum lavage findings of the patients are presented in Table 4. Punctum lavage was negative in 5 of the 37 patients with anatomically patent ostia in the endonasal group, and these cases were considered as functional failures. Punctum lavage was negative in 2 of the 35 patients with anatomically patent ostium in the external group and this was considered as functional failure. The anatomical, functional and overall success rates are shown in Table 5. When both groups were compared in terms of surgical success, no statistically significant difference was found (*p* > 0.05).

**Table 4:** Ostium width, complication status and lavage status data of the groups.

	Endonasal	External	Significance level
Ostium (mm^2^), mean ± SD	14.61 ± 16.66	56.05 ± 60.41	*t* = −3.547
			*p* = 0.001^a^
Complications			
Yes	18	11	*p* = 0.078^b^
No	20	27	
Lavage			
Patent	32	33	*p* = 0.500^c^
Impatent	6	5	

a *t*-test.

b Chi-square test.

c Fisher’s test.

**Table 5:** Functional and anatomical success of surgery.

Ostium	Endonasal		External	
	Punctum lavage (+)	Punctum lavage (−)	Punctum lavage (+)	Punctum lavage (−)
Stenosis (n)	0	1	0	3
Patent (n)	32	5	33	2
Anatomical^a^ success (%)	97.36		92.10	
Functional^a^ success (%)	86.84		94.73	
Overall success^a^ (%)	84.21		86.84	

a *p* > 0.05, Chi-square test.

## Discussion

Anatomical success does not always mean functional success in DCR operations. Functional failure is defined as the persistence of epiphora complaints despite the presence of a postoperative patent anatomical drainage system.^8^ In several studies, despite the presence of anatomically sufficient ostium patency, intracanal synechia, lacrimal pump failure, sump syndrome and the size, location and shape of the ostium are reported to be the reasons of failure.^9^, ^10^, ^11^, ^12^, ^13^ Functional failure may be tolerated by the patient or it may require medical or surgical treatment. Modalities including corticosteroid treatment, punctoplasty, lower eyelid ectropion repair, placement of a silicone tube, upper eyelid blepharoplasty and revision DCR can be used in the case of a functionally unsuccessful DCR.^8^

Several studies have investigated the effect of the ostium size on surgical success and have found inconsistent results. Ezra et al. reported that a wide ostium provided a higher rate of surgical success.^14^ Argin et al. reported that the critical osteotomy size should be 2 × 2 cm.^15^ A wider ostium was created by using mitomycin C during DCR operations, but wider ostium was not associated with an increase in surgical success rates.^16^, ^17^, ^18^ Moreover, a fistula about 6 mm was reported to provide a functional lacrimal system.^9^ In recent years, there have been an increasing number of studies reporting that ostium width is not effective on postoperative success. Iliff created a 10 mm bony window and reported 90% surgical success rate in his study.^19^ Ali et al. reported that 4 weeks after surgery, an ostium measuring ≥8 × 5-mm should be considered to be a satisfactory large size.^20^ Chan and Selva suggested that the ostium width should be determined with consideration of patient features such as lacrimal sac size and location instead of creating a standard-sized ostium in all patients.^1^ In our study, we also found that rhinostomy ostium was significantly wider in the external group. The anatomical success rate was higher in the endonasal group and functional success rate was higher in the external group, although the differences were not statistically significant. Thus, ostium size was not correlated with functional success.

It is reported that the factors such as the use of endonasal or external approach, the procedures applied to the mucosal flap, the use of sutures, antifibrotic use, and the use of perioperative antibiotics may be effective on ostium size.^1^ Creation of a wide ostium facilitates suturing of mucosal flaps in external DCR. Suturing is more difficult in endonasal surgery. Suturing of mucosal flaps reduces scar formation, although no statistically significant difference was found in ostium size between cases with and without flap suturing.^11^, ^21^ In our study, flap suturing was not used in the endonasal group, while the anterior and posterior flaps were sutured in the external group. The smaller final ostium size with endonasal surgery may be due to not performing flap suturing. Similar to the literature, there was no statistically significant difference in terms of ostium stenosis. There are several different factors that cause postoperative ostium stenosis such as wound healing problems and postoperative surgical field infections. In addition, different surgical techniques may also contribute to the postoperative ostium stenosis and this is the one of the limitation of our study. In order to minimize the effect of this situation on the results, the operations of the same surgeons in the relevant department were included in the study.

What is the ideal ostium size that can provide a sufficient postoperative ostium patency? It’s generally accepted that there is no significant shrinkage of the ostium after the 4th week of the operation.^5^, ^6^ The hemostatic and inflammatory phases of wound healing last 1–3 weeks, followed by the remodeling phase, which explains why only minimal changes take place after the 4th week. It is reported that 50%–64% reduction occurs in ostium size by the end of 4th-week of the operation.^1^, ^14^ Thus, postoperative stenosis can be avoided by creating a wide ostium during the operation. More studies should be done about the proper size of the ostium. In our study, ostium sizes were measured at postoperative at least at the 8th week to avoid the effect of the wound healing on the results.

Despite the use of standard operating procedures, ostium sizes were different as each patient had different wound healing process. This may be another limitation of our study. In addition, infections, hypergranulation, and synechia can affect the final ostium size and this decreases anatomical and functional success of the surgery. Inadequate osteotomy and incomplete lacrimal sac marsupialization may be the other factors that affect the ostium size in both techniques.^22^ If necessary for a patent duct or patent ostium, granulation and synechia can be treated. Granulation was found at the ostium level in 8 patients in the endonasal DCR group and in 5 patients in the external DCR group and granulation was surgically excised in 2 patients for the patency of the ostium.

The shape, scar formation, synechia status and location of the rhinostomy ostium have also been investigated for functional success. Shin et al. reported that the shape of the rhinostomy varied depending on the shape of the lacrimal sac and the functional success rate was higher in the” ice scoop” shape group when the shape was categorized into flat, ladle and “ice scoop” shape.^23^ They also reported that other parameters were not effective on functional success. In our study, the circular shape of the ostium was more common than the oval shape.

Various studies compared endonasal and external DCR in terms of the success rates and they reported the success rates as 85%–90% with both methods using appropriate techniques.^24^, ^25^ Endonasal DCR has advantages, including the absence of skin incision, better preservation of lacrimal pump function, rapid healing and less bleeding. External DCR has the advantages of a better exposure and the ease of performance of the surgery. Potential complications of external DCR include bruising, wound infection, and cerebrospinal fluid leaking. Endonasal DCR complications include damage to the nasal mucosa with scar formation, perirhinostomy granuloma, orbital fat prolapse, transient damage of the medial rectus muscle with diplopia, secondary canalicular stenosis, sump syndrome, and adhesions between the ostium and the nasal septum.^26^, ^27^, ^28^ Complications are extremely rare with both techniques. Botek and Goldberg reported in their cadaveric study that complications such as cerebrospinal fluid leak can be prevented with a distance of 25 mm between the anterior aspect of the cribriform plate and the internal common punctum.^29^ It is reported that the risk factors for failure in DCR include a narrow nasal cavity, a thick maxilla, severe anteriorization of ethmoid sinus and a small lacrimal sac.^30^ In our study, minor complications were observed in both groups at similar rates as reported in the literature. In one patient, in the endonasal group, a corneal abscess developed and this resolved with medical treatment. There was no statistically significant difference between both groups in terms of complications in our study.

## Conclusion

Reestablishing the drainage of tears into the nasal cavity is critical in both endonasal and external DCR. Clearly, a patent duct and ostium are required to achieve it. Postoperative stenosis can be prevented by creating a wider rhinostomy during the surgery considering the postoperative shrinkage of the ostium. Moreover, similar to studies performed in recent years, our study found that creating a wide ostium has no effect on postoperative functional success. Further studies in large series may determine the ideal ostium size to reduce the complication risk and functional failure.

## Authors’ contributions

All of the authors declare that they have all participated in the design, execution, and analysis of the paper, and that they have approved the final version.

## Conflicts of interest

The authors declare no conflicts of interest.
